# Promising directions for human-robot interactions defined by older adults

**DOI:** 10.3389/frobt.2024.1289414

**Published:** 2024-04-24

**Authors:** Anastasia K. Ostrowski, Jenny Zhang, Cynthia Breazeal, Hae Won Park

**Affiliations:** ^1^ Media Lab, Massachusetts Institute of Technology, Cambridge, MA, United States; ^2^ Wellesley College, Wellesley, MA, United States

**Keywords:** older adults, social robots, co-design, participatory design, ethnographic decision tree modeling, qualitative analysis

## Abstract

**Introduction:** Older adults are engaging more and more with voice-based agent and social robot technologies, and roboticists are increasingly designing interactions for these systems with older adults in mind. Older adults are often not included in these design processes, yet there are many opportunities for older adults to collaborate with design teams to design future robot interactions and help guide directions for robot development.

**Methods:** Through a year-long co-design project, we collaborated with 28 older adults to understand the key focus areas that older adults see promise in for older adult-robot interaction in their everyday lives and how they would like these interactions to be designed. This paper describes and explores the robot-interaction guidelines and future directions identified by older adults, specifically investigating the change and trajectory of these guidelines through the course of the co-design process from the initial interview to the design guideline generation session to the final interview. Results were analyzed through an adapted ethnographic decision tree modeling approach to understand older adults’ decision making surrounding the various focus areas and guidelines for social robots.

**Results:** Overall, over the course of the co-design process between the beginning and end, older adults developed a better understanding of the robot that translated to them being more certain of their attitudes of how they would like a robot to engage with them in their lives. Older adults were more accepting of transactional functions such as reminders and scheduling and less open to functions that would involve sharing sensitive information and tracking and/or monitoring of them, expressing concerns around surveillance. There was some promise in robot interactions for connecting with others, body signal monitoring, and emotional wellness, though older adults brought up concerns around autonomy, privacy, and naturalness of the interaction with a robot that need to be further explored.

**Discussion:** This work provides guidance for future interaction development for robots that are being designed to interact with older adults and highlights areas that need to be further investigated with older adults to understand how best to design for user concerns.

## 1 Introduction

As more and more technology enters our lives, it is critical for us to be designing these systems with users. Social robots are increasingly being introduced into many different social contexts, including homes, schools, and hospitals, stressing the need for users to be more frequently engaged in social robot design processes. Researchers in human-robot interaction (HRI) have highlighted the promise of social robots assisting older adults, proposing several areas where these technologies could be beneficial (Smarr et al., 2014; Ostrowski et al., 2019b; Abou Allaban et al., 2020; Bardaro et al., 2022) (that have informed the selection of the focus areas for this work). However, older adults are often not included in the design process where researchers, engineers, and designers generate ideas and develop requirements for the technologies. This is often due to stereotypes such as older are unable to use technology (Light et al., 2015; Knowles et al., 2020).

In recent years, there has been more emphasis on engaging users in more participatory design methods, such as co-design, and, specifically, working collaboratively with older adults in the design of social robots. Older adults are essential stakeholders in the design of social robot systems and contribute immense value to the design of these technologies due to their lived experiences and ability to create interactions tailored to their lives (Ostrowski et al., 2021b; Sakaguchi-Tang et al., 2021). Engaging older adults in co-design processes where they partner with researchers and designers empowers them in the design of these technologies as their lived experiences and expertise are valued in the process.

In our work, we explore how older adults’ ideas and future design directions of social robots and design guidelines for social robot interaction change and develop over the course of a year-long co-design process. We analyze older adults’ desired functions and design features from the initial interview and the final reflection interview; from the beginning to the end of the co-design process. We also explore the design guidelines and priorities generated by older adults for the next stage of robot interaction development. Previous works from the larger co-design study have described the overall co-design process (Ostrowski et al., 2021a), presented long-term divergent-convergent co-design guidelines for designing social robots (Ostrowski et al., 2021a), examined the shift in who is called a robot designer (Ostrowski et al., 2022a), explored older adults’ usage of social robots in the home (Ostrowski et al., 2022b), and explored the value of stories in co-design work (Ostrowski et al., 2021b). In this work, we specifically look at the longitudinal change of older adults’ desired features and functions for social robots and their design guidelines from the end of the study. The main contribution from this work is guidance for future robot interaction development generated by older adults. We also highlight specific areas with high promise in older adult-robot interaction and specific areas in need of further investigation and design collaboration with older adults to address concerns surrounding these interactions.

## 2 Background

As we work to design social robots that people will interact with in their homes and other spaces during the course of their day, it is critical for us to design social robot interactions that are created with users.

### 2.1 Designing robots for everyday HRI

We need to think about robot interaction design when we consider everyday interactions and how a social robot will fit into people’s lives. Social robot application research is expansive and has looked at areas from healthcare to education settings (Leite et al., 2013; Sheridan, 2020; Mahdi et al., 2022). Social robots in the home are another important focus area for HRI (Leite et al., 2013), including considering older adults interacting with robots in their home as they age in place (Alves-Oliveira et al., 2015; Mois and Beer, 2020). As the world’s older adult population continues to grow (United Nations Department of Economic and Social Affairs, 2020), there is an increasing need for ensuring older adults are supported holistically while aging in place. It is also critical for more research to be conducted around older adults’ interaction and design preferences since there are many perspectives in the literature on social robots designed to engage with older adults. For example, some research has found that older adults do not want social robots to exhibit any emotional aspects (Thunberg and Ziemke, 2021); while in other research, older adults want the social robot to exhibit these appearances (Eftring and Frennert, 2016). These previous results seem conflicting, however, there is a need to understand the complexity of these results with studies providing more discussion on how to consider these results contextually and the nuances for why people may choose not to engage with social robot interactions and/or designs. Overall, technologies, such as social robots, offer promise for older adults to use in their everyday lives and support their wellbeing (Alves-Oliveira et al., 2015; Mois and Beer, 2020). Considering the development of these technologies that will be used by a variety of older adults and their social and, potentially, healthcare networks, it is critical for us to engage older adults in the design process of social robots to support them in finding ways that these technology systems can best address their needs and to support researchers in understanding how best to approach challenging design areas.

### 2.2 Co-design with older adults in HRI

Co-design (or collaborative design) and participatory design in HRI support deeper engagement with users in the design of robots. Co-design processes support user empowerment in the design process where researchers collaborate with users as partners in the design process (Harrington et al., 2019). When users, such as older adults, engage in co-design processes, they inform the design of technologies, such as social robots, from their expertise and lived experiences (Bate and Robert, 2006; Harrington et al., 2018).

Older adults have engaged in co-design and/or participatory design of social robots in varying ways. For example, older adults have engaged in a co-design processes to design robots through interviews and workshops where they learned about physically assistive and socially assistive robots and created sketches with the research team of their desired robot interactions (Šabanović et al., 2015; Lee et al., 2017; Randall et al., 2018). In a 3 week community-based participatory design study exploring how older adults want social robots to be designed, older adults lived with a social robot in their community space and engaged in card-sorting to express their desired functions for social robots in their community space (Ostrowski et al., 2019a). Co-design processes with older adults designing social robots for emotional wellbeing have employed the dialogue-labs method to support ideation and concept development through structured approaches considering aspects such as the design process, the physical design environment, and the materials used in the design activities (Lucero et al., 2012; Alhouli et al., 2023). Participatory design workshops have also been used in co-design with older adults. For example, in (Fraune et al., 2022), older adults reflected on their technology experiences and social challenges before iteratively designing a robot to solve their social challenges. In another study, older adults partnered with researchers to design a care robot for the home through participatory design workshops, prototyping, interacting with mock-ups, and testing the robot in the home (Eftring and Frennert, 2016). This work gathered user requirements around how the robot should interact with older adults in the home, including details on the embodiment (e.g., maximum height, appearance) and interactions (e.g., picking up objects, assisting with exercises). Design scenarios can also be incorporated into participatory design activities to support older adults in considering how robot interactions could be designed for various contexts, including investigating how various types of robots could be used in design scenarios (Thunberg and Ziemke, 2021). Participatory design sometimes includes techniques that allow older adults to prototype on the robots (as seen in this study and discussed in (Ostrowski et al., 2022a)). In an adaptation of participatory design, termed Situated Participatory Design, older adults designed and tested robot interaction scenarios in their homes through experiences with the robot, and Wizard of Oz techniques to simulate interactions (Stegner et al., 2023). These examples of co-design and participatory design with older adults demonstrate how older adults can be more deeply engaged in the design process and how their lived experience can be valued in the design of social robots. They also call for more longitudinal studies to support the robot development process in partnership with older adults (Thunberg and Ziemke, 2021). Overall, co-design and participatory design are less common methods when engaging older adults in design processes and these methods are not commonly used within HRI (Björling and Rose, 2019). Our work demonstrates how older adults’ experience in a co-design process can support them in creating design guidelines for social robots and how their thoughts and opinions may shift overtime in this process.

## 3 Methodology

Embedded in a year-long co-design process, we conducted interviews and a design guideline generation session with older adults to understand their ideas for robot interaction in their everyday lives. As discussed in this paper, the initial and reflection interviews delved into discussing the core domains of impact. The design guideline generation session was a collaborative session with older adults to identify key areas of development and improvement moving forward with social robots. This section outlines the protocols for these sessions and the analysis process for the different types of data.

### 3.1 Overview of co-design process

**FIGURE 1:**
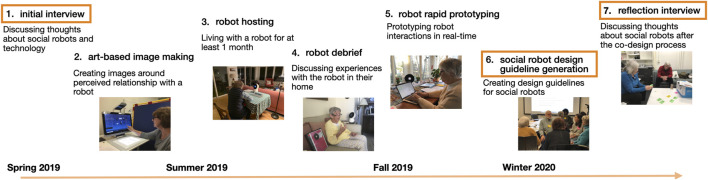
Year-long co-design process with seven sessions detailed. The sessions that are the main focus of this paper (initial and reflection interviews, and design guideline generation sessions) are highlighted in boxes.

We conducted a year-long seven stage co-design process collaborating with older adults to understand the key focus areas that older adults see promise in for older adult-robot interaction in their daily lives (Ostrowski et al., 2021a) (Figure 1). The co-design process provided opportunities to be expansive in their idea generation, creating new areas for robot use, and also provided scoped exploration of seven focus areas: connecting with others, medical adherence, memory assistance and monitoring, exercise and physical therapy, body signal monitoring, emotional wellness, and financial management (informed by previous research in HRI). Over the course of the year, we engaged in multiple different activities including interviews, art sessions, interactive prototyping, and a design guideline generation session (for more details on the overall process, please refer to Figure 1; Ostrowski et al. (2021a)). In this work, we specifically explore three of these sessions: the initial interview, the design guideline generation session, and the reflection interview. This work describes robot-interaction priorities identified by older adults, tracking these priorities from the beginning to the end of the co-design process.

### 3.2 Participants

In the year-long co-design process, we collaborated with 28 older adults between 70 and 94 years of age (mean: 79.5, std: 7.8; female N = 15) from three states in the United States. Participants chose whether they would like to complete their sessions at the MIT Media Lab or their home based on their preference. Participants who were not located in the state of Massachusetts completed their sessions remotely through video calls. The co-design study was approved by the Institutional Review Board (IRB) and all participants volunteered to participate, completing an IRB approved consent form. For the three activities that we focus on in this paper, all participants participated in the initial interview, 79% participated in the design guideline generation session, and 75% participated in the reflection interview. Those who could not attend the design guideline generation session were unable to attend due to travel or health-related issues. The COVID-19 pandemic was just starting while we were conducting the reflection interviews, therefore, some participants did not complete this session due to the start of the pandemic or lack of online access. Additionally, due to the COVID-19 pandemic, some of the reflection interviews were conducted through video call.

### 3.3 Interviews in co-design process

The interview protocols for the initial interview and reflection interview were structured using an ethnographic decision tree modeling approach (Gladwin, 1989). The structure for the two interviews was similar with some variations based on the stage of the process. The initial interview began with an opening providing an overview of the co-design process and introduction to the seven focus areas: connecting with others, medical adherence, memory assistance and monitoring, exercise and physical therapy, body signal monitoring, emotional wellness, and financial management. The participants were then asked to discuss their everyday lives, interactions with technology, and their initial thoughts on social robots and the roles they see social robots potentially playing in their lives. This section was only asked in the initial interview. At the beginning of the reflection interview that occurred after the design guideline generation session, the interview started with the participant reflecting on the design guideline generation session.

After these beginning sections, the interview progressed into looking at each of the focus areas specifically. For each focus area, for the initial interview, participants were asked (1) what they thought of when they heard the focus area, (2) if the focus area is part of their life and if so, how do they engage in the focus area, (3) their initial thoughts for a social robot assisting with this area, and (4) if such a social robot interaction would fit into their lives. These questions were only asked in the initial interview. In the reflection interview, each area was revisited and participants were asked their thoughts on social robots assisting in the focus area and how it may fit into their lives.

For both interviews, the interviewer then asked participant’s thoughts about specific actions that a robot could do in each focus area. For example, for *medial adherence*, one interaction asked about was if a person would like the social robot to remind them when to take their medicine. Another example for *connecting with others* was if the person would like the social robot to remind them to call or talk to friends and family.

**FIGURE 2:**
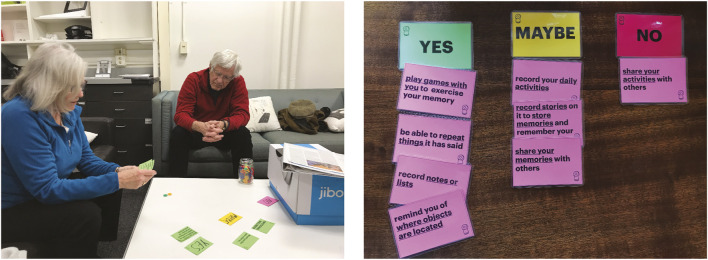
On the left, older adults participating in the reflection interview card sorting; on the right, participant arranged cards for the memory focus area, sorted into “yes [I want this function]”, “maybe [I would want this function]”, and “no [I do not want this function]”.

This part of the interview was formatted differently for the two interviews because of co-design participant input. In the initial interview, the participant and interviewer completed this section verbally. Participants mentioned that it was a bit tedious to go through these questions verbally. Therefore, based on this suggestion, for the reflection interview, the researchers turned these questions into physical cards that the participants could sort based on their preferences for the various interaction types (Figure 2). For both interviews, participants were asked to explain their preferences for or against the various interactions. Another modification informed by participant feedback was removing the financial management category for the reflection interview. In the initial interview, there was a majority negative reception to the category and the category was removed from the subsequent activities.

After participants completed this section stating their preferences for some interactions that may be included in the focus areas, they were asked how they felt about a social robot in this focus area overall and any concerns and/or benefits they could see of a social robot in this focus area. As a closing for the interview, participants lastly discussed the most intriguing and most appalling or shocking scenario, barriers for having a social robot in their home, and benefits of having a social robot. For the reflection interview only, participants also reflected upon the overall co-design process. The full protocol for both interviews can be found in the Supplementary Materials.

#### 3.3.1 Interview analysis: Adapted ethnographic decision tree modeling

Ethnographic decision tree modeling is a qualitative method where through interviews researchers explore decision criteria and motivation (Gladwin, 1989). The methodology is grounded in participants’ expertise, lived experiences, and beliefs that their decision making is based upon (Gladwin, 1989; Roth and Botha, 2009), aligning with values supported by co-design. In this paper, we used ethnographic decision tree modeling as a foundational analysis technique to explore older adults’ decisions for or against social robot interactions across the focus areas. We adapt the ethnographic decision tree modelling process to explore the reasons that are motivating older adults decision making, restructuring the tree to support visualizing these decisions. We also expand the methodology by the creation of meta-analysis and final trees (more information below), providing an overall scope of the older adults’ decision making around robot interactions. In this way, we align with the phases of exploration and model development common in ethnographic decision tree modeling. We do not engage with the model testing phase as this is one aspect of future work.

A set of Ethnographic Decision Model Trees were created from each participant interview to understand the participants’ decision making process while they conceptualize their ideal social robots and interpret whether participants were receptive or against seeing focus area interactions in a robot. The decision model trees were created in three stages. All decision model trees are based on participant transcripts that were created from audio recordings of the interviews. Two researchers reviewed the transcripts and iteratively developed the decision model trees.

**FIGURE 3:**
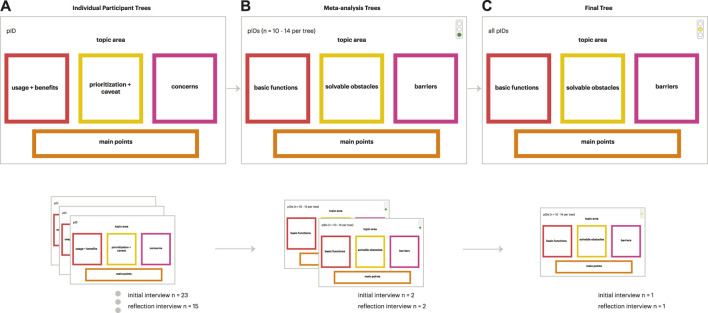
Iterative process of developing model trees beginning with individual trees **(A)**, meta-analysis trees **(B)**, and the final tree **(C)**. The numbers of trees per stage for the initial interview and reflection interview, respectively, are shown on the bottom.

In the **first stage**, we created a set of decision model trees for each participant interview, which consists of seven focus areas: medication adherence, body signal monitoring, memory, emotional wellness, connecting with others, exercise, and financial management. As a decision model tree is created for each focus area, each participant set typically consists of seven model trees. Each decision model tree consists of three branches (Figure 3A). Under the first branch, “Usages + Benefits,” are the functions that the participant would readily accept in a social robot. In the second branch, “Prioritization + Caveat,” are functions that were rejected by the participant, or treated with hesitation. Under the third branch, “Concerns,” are broader concerns that the participants have about social robots that contribute to their hesitation or rejection of certain functions. After each model tree was completed, the main emphasis of the participant was summarized in a “Main Points” box, recapitulating the general outlook that the participant has towards social robots and the focus areas and specific functions to which they are the most receptive or resistant.

After the participant level decision model tree sets were completed, for **stage 2**, the decision model trees are organized into groups of 8–14 to construct composite Meta-analysis Decision Model Tree sets for a broader analysis of the participants’ shared attitudes towards the focus areas and individual functions. As a Meta-analysis Decision Model Tree is constructed for each category, each decision model tree set contains seven individual model trees (Figure 3B). Each decision model tree has three branches. The first branch, “Basic Functions,” include functions that were readily accepted by the participants. The second branch, “Solvable Obstacles,” are functions that were accepted by some, but not all participants, or treated with hesitation. The third branch, “Barriers,” includes the broader concerns that participants have about technology that could underlie their rejection of certain functions. The pertinent opinions that the participants had for each category are summarized in the “Main Points” box. The participants’ acceptance for each category is ranked using a “Traffic Light System.” The Traffic Light System is a color coding system that demonstrates the participants’ degree of acceptance for each category and is based on the traffic light systems in the United States where the research was based. Each category is marked with a circle colored: (1) green, denoting that participants were very receptive and enthusiastic for this category; (2) yellow, stating that participants hold some reservations for this category; or (3) red, meaning that participants do not find this category useful, or are reluctant to use the robot for functions in the category.

In **stage 3**, the two Meta-analysis Decision Model Tree sets are consolidated to create the Final Analysis Decision Model Tree set, which captures the themes that emerged from the participants as a full group (Figure 3C). The branches of the trees are the same as those of Meta-Analysis trees. Similarly, the participants’ degree of acceptance for each category is ranked by the Traffic Light System. This model tree analysis process was the same for the initial and reflection interviews (with the exception of excluding the financial management focus area for the reflection interviews as discussed above).

### 3.4 Design guideline generation session in co-design process

**FIGURE 4:**
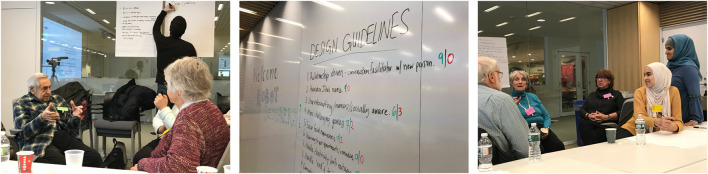
Older adults and researchers during the design guideline generation sessions where older adults generated design priorities and voted on design guidelines for social robots.

The design guideline generation session was modelled from Gonzales and Morrow-Howell (2009)’s focus groups with inspiration from resources including “Research from the Periphery: Resources for Community-Led Action Research at Instituto Banco Palmas” (MIT CoLab, 2015), “DiscoTech” Zine (Digital Detroit Justice Coalition, 2012), “Design Justice In Action” (Design Justice Network, 2018), and “Principles for Design Justice” (Design Justice Network, 2016). The goal of this session was for the co-design participants to generate the design guidelines for the future development of robot interactions. We engaged in two main activities in the design guideline generation session in collaboration between the larger research team (12 researchers) and older adults (Figure 4). The first activity asked participants in small groups to generate a list of design priorities for their social robots. We encouraged the small groups to generate at least 10 design priorities while reflecting on their co-design experience and future desires for the technology. In the second activity, participants voted on the design priorities generated by all the small groups. The session lasted 3 hours long. The design priorities generated and voted upon by the participants were categorized by researchers to reveal the overall design guidelines for the future of the robot interaction design.

## 4 Results

### 4.1 Initial interviews

#### 4.1.1 Individual model trees

The functions for the various focus areas were organized as usage and benefits, prioritization and caveat, and concerns for each individual participant model tree.

**FIGURE 5:**
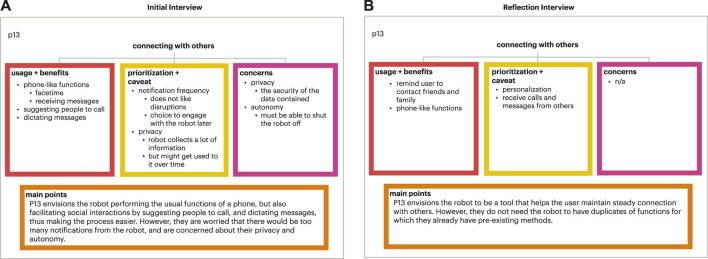
Example of individual tree set. **(A)** Initial interview individual tree for p13 for the connecting with others focus area; **(B)** Corresponding individual tree for p13 for the reflection interview.

The usage and benefits branch included functions that were most readily accepted by the participant because they were seen as the most useful (example show in Figure 5). The functions under this branch generally were: (1) administrative - setting reminders, providing and storing information, and phone-like functions such as text dictation and video calling; (2) motivational - offering suggestions and encouragement, and keeping users accountable by setting goals and checking-in; or (3) light entertainment - telling jokes and fun facts. For the prioritization and caveat branch, functions were those rejected by the participant, or treated with hesitation. However, the reasons for the participant’s hesitation or rejection could be abated with time, stipulations, and/or customization, allowing the participant to be more accepting of the functions in the future. The reasons and solutions for the participant’s hesitation could be the following: (1) personalization - the participant would like to customize the robot to their needs. For example, participants could opt in/out of the functions they want, or customize the frequency of reminders; (2) othering—the participant does not want the function personally, but could see it being useful for others; or (3) changing skepticism—the participant may grow to accept certain functions as they become more comfortable with the robot. For example, a participant may not want to use mood tracking at first due to their lack of trust in the robot, but could envision using it in the future when their trust in the robot increases. Lastly, the concerns branch included sentiments shared by the participant that were reminiscent of greater concerns for technology. These concerns and skepticism contribute to the participant’s hesitation and rejection of certain functions. Unlike the reasons for hesitation described in the previous branch, these concerns are broader and more abstract. Thus, they cannot be addressed with stipulations, but rather require a change in the user’s perception of technology based on how technologies address these concerns. Some common concerns were: (1) losing autonomy, such as becoming overly dependent on the robot and consequently losing certain capabilities from disuse; (2) privacy and security of user data; (3) the robot’s lack of humanness, which inhibits the robot from providing the same quality of care as human connections; or (4) the robot’s lack of expertise in areas such as medicine, and is therefore unqualified to offer suggestions and advice in those fields.

#### 4.1.2 Meta-analysis and final model tree sets

**FIGURE 6:**
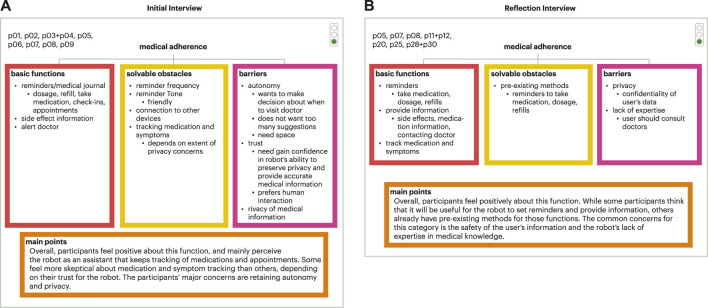
Example of meta analysis tree set. **(A)** Initial interview meta analysis tree for a portion of the participants for the medical adherence focus area; **(B)** Corresponding meta analysis tree for the reflection interview.

The meta-analysis model tree sets and final model tree sets were both organized with three branches (basic functions, solvable obstacles, and barriers), including a main points section emphasizing key takeaways on older adults’ decision making (example show in Figure 6). The results from the final model tree were similar to those in the meta-analysis trees. The full set of initial final tress can be found in the supplementary materials. Therefore, results for both are discussed in this section.

In the basic function branch, the functions were those that were readily accepted by most of the participants. Across the focus areas, these functions could be categorized as: (1) administrative, such as setting reminders and tracking and visualizing data, (2) motivational, such as offering encouragement and coaching, and (3) light entertainment, such as telling jokes and fun facts. These results are similar to what was seen in the individual trees. The solvable obstacles branch was modified from the personalization branch in the individual trees. The solvable obstacles branch included functions that were accepted by some, but not all participants. The functions that were rejected by participants could be accepted with time or stipulations. The reasons and solutions for the hesitation towards certain functions felt by participants are: (1) customization/personalization - the participants would like to customize the robot to their needs, such as when and with whom the robot should share data; (2) pre-existing methods - participants may already have methods in place for certain functions, such as medication refill reminders, and feel that having those functions in the robot would be redundant; or (3) othering—participants may feel that certain functions may not be useful for themselves, but could see them being helpful for others. For example, some think emotional support functions may be more useful for those living alone. Barriers included the broader concerns surrounding technology that could underlie the hesitation and reluctance that participants feel towards certain functions. Some common concerns are (1) autonomy; (2) privacy and security of user data; and (3) the robot’s lack of humanness.

In this iteration of the trees, we introduced the traffic light system (as described in Section 3.3.1). For the meta-analysis model trees and final model tree sets, the focus areas of medication adherence, memory, and exercise were all categorized as green, meaning well-accepted and a clear area for future robot interaction development. Emotional wellness, body signal monitoring, and connecting with others were categorized as yellow, indicating promise for this space for robot interaction development but there needs to be more exploration in this space to understand the nuances for development. Financial management was the only focus area categorized as red as older adults were opposed to a social robot assisting in this area.

### 4.2 Reflection interviews

#### 4.2.1 Individual model trees

The functions for the various focus areas were organized as before with these three branches, usage and benefits, prioritization and caveat, and concerns, for each individual participant model tree. For usage and benefits, the functions generally were categorized as: (1) administrative and (2) light entertainment. For prioritization and caveat, a participant’s hesitation towards certain functions could be due to (1) personalization, (2) pre-existing methods, (3) othering, or (4) potential future usage. Potential future usage described when the participant may not need a function at the current moment, but could envision it being useful in the future when they would need more assistance with memory and health. For the last branch, the concerns held by the participant could be (1) autonomy, (2) privacy and security of data, (3) the robot’s lack of humanness, or (4) the robot’s lack of expertise.

#### 4.2.2 Meta-analysis model trees and final model tree sets

As with the initial interview analysis, the meta-analysis model tree sets and final model tree sets were both organized with three branches (basic functions, solvable obstacles, and barriers), including a main points section emphasizing key takeaways on older adults’ decision making. The results from the final model tree were similar to those in the meta-analysis trees. The full set of reflection final tress can be found in the supplementary materials. Therefore, results for both are discussed in this section.

For basic functions, the functions could be categorized as (1) administrative and (2) light entertainment. For solvable obstacles, the participants’ hesitation towards certain functions could be due to (1) personalization and/or (2) pre-existing methods. Some common barriers shared by participants were (1) autonomy, (2) privacy and security of user data, (3) the robot’s lack of humanness, and (4) the robot’s lack of expertise.

By the traffic light system, the focus areas of medication adherence, memory, and exercise were all categorized as green and a promising area for robot interaction development. The focus areas of emotional wellness, body signal monitoring, and connecting with others were categorized as yellow, demonstrating promise but in need of more investigation to design these interactions properly with older adults.

### 4.3 Comparing initial and reflection interviews

**FIGURE 7:**
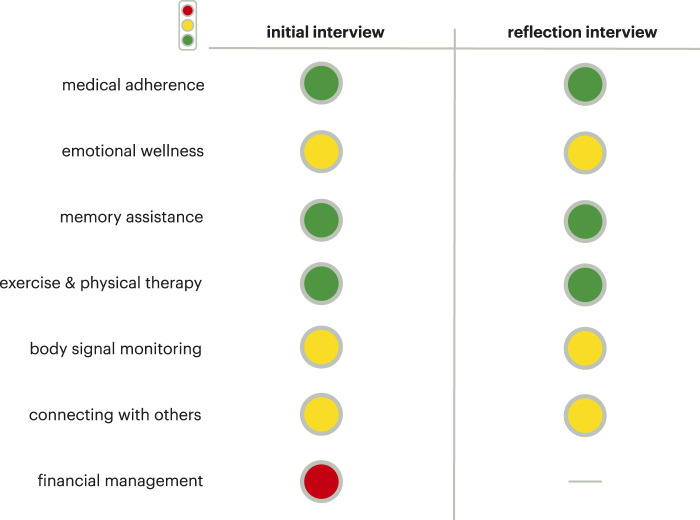
Summary of traffic light system results demonstrating no change in the overall categorization of the functional categories (except for financial management that was removed because of the extreme negative sentiment after the initial interviews).

Between the initial and reflection interview ethnographic decision tree analysis, there were differences and similarities, demonstrating how through the co-design experience older adults changed their perspectives toward focus areas or remained with their original perception. With regard to older adults’ sentiments, for both rounds of interviews, the participants were more accepting of administrative functions such as reminders and scheduling, and less open to what were seen by older adults as more invasive functions such as tracking and emotional support. The traffic light system results are the same for the initial and reflection interviews (Figure 7). This means that the participants’ overall sentiments towards the functional categories did not change as a result of their experience with the social robot, Jibo, or the change in interview format. The greater concerns regarding technology that underlie the reservations that participants had for certain functions remained constant throughout both interviews. This shows that experience with the robot did not alleviate those concerns, and that a general shift in the attitude towards robots and technology may need to occur for these concerns to be addressed. A key difference seen between the initial and reflection interviews was in how older adults delivered and discussed their opinion of the various focus areas. In the co-design process, participants lived with a Jibo social robot for at least a month period (though some participants chose to live with the robot longer), allowing participants to get a better understanding and lived experience of what it could mean to have a robot in their home. Over the course of this experience, the participants developed a better understanding of the robot and, therefore, they were more certain of their attitudes towards the functions. We saw this change of certainty between the initial and reflection interviews.

### 4.4 Design guideline generation session categorizations

The design guideline generation session happened right before the reflection interview and provides an understanding of participants’ design priorities at the end of the co-design process. The design guidelines from the session were categorized into 10 areas: assistant-like tasks, operations, role and personality, ethics, learning, social connection, integration, health and safety, and programming (Table 1). Older adults desired more assistant-like abilities with social robots able to assist with reminders, storing contact information, organizing older adults’ calendars, and general memory assistance. For operations, older adults detailed design guidelines that could improve the general usability of the social robot, including the robot having multiple accounts, being easily cleaned, being able to handle a WiFi or electrical outage, providing closed captions, having the ability to modify robot speech speed, providing multiple ways to get the robot’s attention, and having physical proactivity that can be personalized. In another design guideline category, older adults emphasized the need for integration with other devices with the social robot being able to share information with other devices, control the function of other devices (e.g., lights, doors, etc.), and have seamless function across devices. Older adults also desired the ability to program their social robot in their home. This was possibly inspired by the robot rapid prototyping session in the co-design process that occurred before the design guideline generation session.

**TABLE 1:** Categorized design guidelines and older adults’ acceptance of the guidelines with their corresponding votes for guideline category.

Design guideline category	Average percentage (%)
Assistant-like tasks (e.g., reminders of appointments, note-taking, etc.)	90
Operations (e.g., closed caption option, memory of previous experiences, etc.)	88
Role and personality (e.g., empathetic personality, relationship drive, etc.)	87
Ethics (e.g., trust and privacy features, data security, etc.)	85
Learning (e.g., teach a language, reading audiobooks, etc.)	84
Social connection (e.g., video call functionality, socially aware and will not interrupt, etc.)	81
Integration (e.g., connected to multiple devices, transfer information between devices, etc.)	80
Healthcare and safety (e.g., medication reminders, medical alarm, etc.)	79
Programming (i.e., user can program the robot)	67

Older adults also mentioned design guidelines around the role and personality of a social robot, such as the robot being empathetic and companion-like, being relationship-driven, and having more interpretable facial expressions and human mannerisms. As seen in the barriers and concern areas of the model trees, ethics was an important area of the design guidelines, ensuring data security, trust, and privacy features that the user can control. Learning was also valued by older adults and they wanted the social robots to support their learning through ways such as learning a new language, answering questions through search engines, reading audiobooks, and supporting lifelong learning. Another area of importance was social connection with older adults wanting social robots to have video call functionality, notify them of new photos of their family that have been posted, and start/prompt conversations with family and friends. They noted the importance of the robot to be socially aware with manners and not interrupt people. Healthcare and safety was an important area as supported in the decision model trees. Older adults wanted the social robots to be equipped with medical sensors to monitor their health and safety, including having emergency functions to alert emergency services and family in case of an emergency, medication reminders, and overall diet and exercise recommendations. Older adults created design guidelines that captured a range of design aspects from operations and basic functions of the robot to specific robot interaction design features.

## 5 Discussion

**FIGURE 8:**
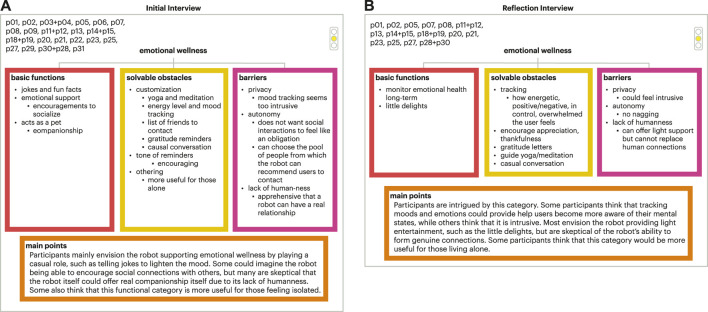
Example of final tree set. **(A)** Initial interview final tree for the emotional wellness focus area; **(B)** Corresponding final tree for the reflection interview.

**FIGURE 9:**
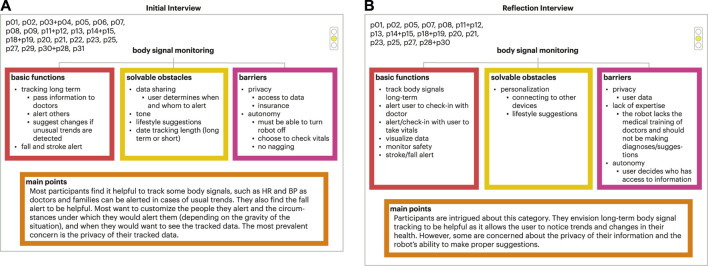
Final tree set for body signal monitoring. **(A)** Initial interview final tree; **(B)** Corresponding final tree for the reflection interview.

**FIGURE 10:**
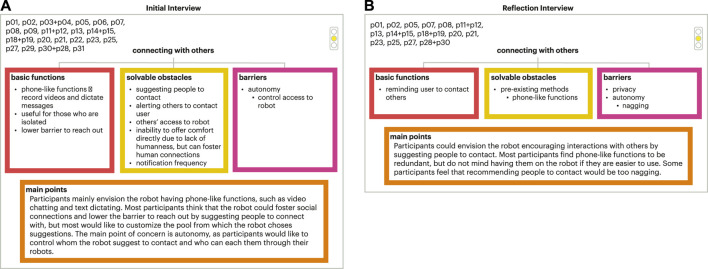
Final tree set for connecting with others. **(A)** Initial interview final tree; **(B)** Corresponding final tree for the reflection interview.

Older adults expressed their opinions and directed future design directions for social robots in their lives. Throughout the co-design experiences, older adults gained a better understanding of social robots that informed their generated design guidelines and thoughts for social robots across the seven focus areas, demonstrating the value of the lived experience with the technology and the larger co-design process as older adults collaborated with the research team. Overall, older adults were accepting of transactional functions seen in both the final set of the decision model trees and design guidelines. Focus areas that involved sensitive information, monitoring, and/or tracking were less accepted and/or desired by older adults in our study. This was expressed through older adults’ concerns around autonomy, privacy, and the naturalness of the interaction. While there was high acceptance for focus areas of medical adherence, memory assistance, and exercise and physical therapy, the focus areas of connecting with others, body signal monitoring, and emotional wellness were less accepted in part due to older adults’ concerns around autonomy, privacy, and naturalness of the interaction, signalling the need for more research in this space to understand how these should be developed, if at all, in partnership with older adults. For more details on these three focus areas and older adults’ concerns, refer to Figures 8–10. In this section, we explore future areas of exploration for social robots as defined by our older adult collaborators informed by current HRI research and reflect upon components related to social robot transparency that influence older adults’ experiences with social robots.

### 5.1 Older adult defined areas for exploration

Older adults expressed varying levels of support for the focus areas, outlining future directions for these focus areas. Table 2 highlights potential future investigations for the focus areas that older adults were accepting or potentially accepting for, noting HRI work that is beginning to explore this space or provide avenues for HRI researchers to integrate these areas into robot interaction design. By drawing attention especially to the areas where there is potential for the interaction though not quite accepted at the moment, we hope to promote greater research and collaboration with older adults on these specific function categories. We do not include financial management in this discussion as older adults were largely opposed to this interaction with social robots.

**TABLE 2:** Key areas for further investigation for focus areas as identified by older adults and relevant work that is engaging in these spaces.

Focus area	Proposed directions of research	Relevant research exploring areas
Medical adherence	• Exploring how social robot interactions in this area can interact with already existing ways that older adults manage their medical health	Bardaro et al. (2022); Anghel et al. (2020); Wilson et al. (2020)
	• Investigating trust within interactions with older adults that surround medical health	
	• Understanding the role of social robots within the medical healthcare network with regards to expertise and how that is communicated to older adults	
Emotional wellness	• Investigating the variety of roles that social robots can take when engaging with someone in this area	Abdollahi et al. (2022); Jeong et al. (2023)
	• Exploring patterns of customization and personalization that will support how these systems can be effective for older adults	
	• Understanding how to balance leveraging technologies that provide information about emotional state and intrusiveness to the user	
Memory assistance	• Investigating and designing interactions that promote older adults’ independence *versus* dependency on the robot	Lima et al. (2021); Kubota (2023); Lee et al. (2023); Van Maris et al. (2020)
	• Exploring how memory assistance can be both functional (i.e., object location, reminders) and personal (i.e., photo books, personalized memory reminders)	
Exercise and physical therapy	• Exploring patterns of customization and personalization that will support how these systems can be effective for older adults depending on strategies that work best for each older adult	Shao et al. (2019); Rea et al. (2021); Antony et al. (2023)
	• Understanding the role of social robots within the healthcare team network with regards to expertise and how that is communicated to older adults	
Body signal monitoring	• Understanding the role of social robots within the healthcare team network with regards to expertise and how that is communicated to older adults	Chen et al. (2023); Nasr et al. (2021)
	• Exploring how multiple systems and devices can be integrated together (i.e., smart watch, smart speaker)	
Connecting with others	• Exploring patterns of customization and personalization that will support how these systems can be effective for older adults depending on strategies that work best for each older adult	Fan et al. (2021); Coghlan et al. (2021)
	• Understanding the unique capabilities that social robots offer for this focus area without being redundant to existing technologies (i.e., phones)	
	• Supporting easier use of existing technologies (i.e., cellphones) through voice technology or speech-to-text displays	

There are several proposed directions of research across the six focus areas that older adults were either acceptable to or hesitant towards. Many of these important research areas touch on integration of systems, whether that is the healthcare system connecting together older adults, care takers, doctors, physical therapists, and other members of their healthcare team or multiple devices integrating together for an interaction such as a smart watch or cellphones with the social robot (Cresswell et al., 2018; Hung et al., 2019; Van Wynsberghe, 2020; Lee et al., 2023). This also calls for researchers to consider what technologies and other non-technology solutions work well already in older adults’ lives and how they can integrate and pair social robot technologies with these already existing solutions (Boada et al., 2021). With regards to interactions that require health data or data that could be used for healthcare applications, older adults expressed a need for expertise and how not trusting the robot’s expertise could make the interaction untrustworthy, stressing the need for researchers to explore if the robot should take the role of an expert, how to design transparent interactions on the level of the robot’s expertise, and how the role the healthcare team takes in a network with a social robot as a tool (Cresswell et al., 2018; Felzmann et al., 2019; Aymerich-Franch and Ferrer, 2022). Developments in customization and personalization for these systems hold promise for older adults to gain the most benefits, especially as these systems can be tailored to older adults’ preferred exercise styles, communications styles, and other preferences supporting more custom engagements and support (Umbrico et al., 2020; Coghlan et al., 2021; Khosla et al., 2021; Jeong et al., 2023). This approach could also help solve the incongruencies that may exist between various research studies. For example, with regards to the emotional wellness focus area, previous literature has found that older adults would not like the robot to engage with them through emotional aspects or aspects that could be interpreted as companionship (Thunberg and Ziemke, 2021), while others have found that older adults would support these aspects (Eftring and Frennert, 2016). Our work saw both aspects of this. In the interviews, emotional wellness was discerned as not quite accepted and we were able to identify the barriers that may prevent people from being open to this interaction on a robot. In the design guidelines generated by older adults, role and personality, including an empathetic personality and relationship driven personality, were identified as areas that need to be further explored and supported perhaps through personalization to accommodate various preferences. In order to understand the incongruencies between studies, researchers should apply the lens on how to understand these results contextually and what the confounding variables are, in order to address the wide spectrum of older adult preferences and personalization strategies. The works highlighted in Table 2 are not representative of all works that are engaging in the six focus areas but provide directions for research that is being done in these areas and around some of these proposed directions of research. It is important to note that the works cited do not address all proposed directions of research and more work is required especially around investigating the fine line between data collection, intrusiveness, and transparency.

### 5.2 Considering older adults’ interaction experiences related to transparency

Ethics, including trust and privacy features, and data security were front of mind for many participants as seen through the decision model trees and the design guidelines, stressing the importance of investigating ethics and older adults’ concerns related to ethics when designing social robots. Older adults’ concerns around robots and their transparency in interactions focused on privacy, autonomy, and lack of naturalness in our work. Older adults’ concerns related to privacy, including who has access to the data on the robot, if the data collected is secure on the robot, and the intrusiveness of the robot and data collection, building on previous work around privacy in social robots (Sharkey and Sharkey, 2012; Lutz et al., 2019; Wangmo et al., 2019; Belk, 2021; Boada et al., 2021). Autonomy concerns and features to address autonomy concerns voiced by older adults included the ability to turn the robot off and having the ability to program the robot themselves, demonstrating how older adults were generating design features that enabled them to have greater autonomy in their interactions with robots (Coghlan et al., 2021; Ostrowski et al., 2022a). In another dimension of autonomy, older adults were concerned about their own independence and autonomy and how they did not want to become reliant on the robot, echoing findings from previous research when older adults consider using new technologies (Sharkey and Sharkey, 2012; Belk, 2021; Boada et al., 2021; Coghlan et al., 2021). Previous works have also emphasized the need for robot technologies to support older adults’ freedom, control, and independence (Frennert, 2016). As we’ve seen in other areas where robots are being proposed as new tools and solutions (Sharkey and Sharkey, 2012; Belk, 2021), older adults were adamant that social robots should not replace people, especially thinking about those in the their healthcare and social networks (Wangmo et al., 2019; Belk, 2021; Boada et al., 2021). This was connected to the lack of naturalness in interactions that older adults focused on especially in their design guidelines such as wanting a social robot to mirror social conversational norms to make the interaction more natural and intuitive. Lack of naturalness has been identified as a barrier in interactions stressing the continued importance of increasing the fluency and naturalness of interactions with robots (Edwards et al., 2019). These are a few ethical and transparency dimensions that older adults specifically mentioned in the interviews and design guideline generation session. Researchers need to consider these ethical and transparency dimensions and other ones that will continue to develop as more interactions are designed for social robots in these areas (Van Wynsberghe, 2020). These could include greater focuses and interrogations of emotional deception (Van Maris et al., 2020), emotional attachment (Van Maris et al., 2020; Boada et al., 2021), interaction deception (Wangmo et al., 2019; Danaher, 2020; Boada et al., 2021; Sharkey and Sharkey, 2021), and manipulation (Belk, 2021; Fronemann et al., 2022), to name a few. While not specifically mentioned by older adults in this work, it is essential for researchers and designers to interrogate the justice of these devices, considering distributive justice (i.e., who has access to robots and the benefits of robots?), politics of technosolutionism (Morozov, 2013), social equality, and ecological sustainability (Boada et al., 2021).

### 5.3 Limitations and future work

Our work has illuminated several exciting and critical directions for social robot research. The work also has limitations to be noted when exploring future directions. Due to the evolving nature of co-design work that allows participants to help direct the research process and activities, we modified the reflection interview protocol based on participant feedback from the initial interview protocol to include a card sorting exercise. The card sorting exercise encompassed the same questions asked in the initial interview, though it did lead to often more concise responses to that portion of the interview. This was balanced out by the open-ended questions that surrounded the card sorting exercise. Participants voiced the value of this format overall.

The participants we collaborated with live in the United States with the majority of participants living in areas with many colleges and universities where it may be common to engage in research projects. The geographic area where the majority of participants were from may have contributed to our participant sample being all-white. Participating in social robot studies often requires participants to have WiFi access in their home, such as the case of our study where if participants did not have WiFi access they could not participate. This contributes to the need for more attention and conversations around how to engage those that do not have WiFi access or other such resources and the ethical considerations of not engaging these populations and basing research in this space solely on populations who have access to WiFi and other such resources (Wu et al., 2015; Veinot et al., 2018; Hargittai et al., 2019). Due to these limitations, future work when exploring and co-designing social robot interactions should work with more older adults that represent varying geographic areas and ethnic, economic, and cultural backgrounds.

In addition to doing future work to engage more participants to express their opinions and lead future social robot interaction design, future work should engage further with the areas that were identified as “yellow” in the traffic light system to work with older adults to better understand how to design these systems. This may include investigating the trend of “othering” that we often saw (also seen in Deutsch et al. (2019)), when older adults could see perceived benefit for this interaction not for themselves in this moment but for other people or for themselves later in life, to understand how opinions to these focus areas may change as older adults’ lives change. Future work can also test the decision models generated in this work (the next step in the ethnographic decision tree model process, model testing (Gladwin, 1989)) and explore the barriers to usage that older adults identified, including exploring how robot interaction features can be designed to foster older adults feeling empowered around privacy, autonomy, and trust.

## 6 Conclusion

This paper explores older adults’ perspectives towards specific focus areas of social robot interactions over the course of a year long co-design process informed by interviews and a design guideline generation session. While there was no overall change of opinions towards the focus areas, older adults articulated their thoughts and opinions with more detail as the co-design process progressed, identifying key areas of development for social robot interactions and areas of concern. These findings contributed to proposed directions of research across six focus areas for future investigations into social robot interactions around medical adherence, emotional wellness, memory assistance, exercise and physical therapy, body signal monitoring, and connecting with others that robot designers and researchers should engage with to support older adults’ engagement with and usage of social robots. We also outline critical ethical dimensions, including transparency, intrusiveness, privacy, and social robot implementation, that need to be further investigated to promote responsible design of social robot interactions. Through this work, we encourage designers and researchers to address these research directions in collaboration with older adults to create social robots designed and supported by older adults.

## Data Availability

The datasets presented in this article are not readily available because the gathered participant transcripts are not shareable. Requests to access the datasets should be directed to akostrow@media.mit.edu.
