# Supplementary material for: Promising directions for human-robot interactions defined by older adults
Source: Front Robot AI. 2024 Apr 24;11:1289414. doi: 10.3389/frobt.2024.1289414 (PMC11077273; doi:10.3389/frobt.2024.1289414)
Supplement: Supplementary file 1 [file DataSheet1.PDF]

Supplementary Material

1 INTERVIEW PROTOCOLS

1.0.1 Initial Interview Protocol

Opening

Thank you for coming today to participate in our interview. Today, we will be exploring your opinions and preferences around social robots in interactions including:

- Medication Adherence
- Emotional Wellness Coaching/Mood Sensing and Intervention
- Connecting with Others
- Exercise / Physical Therapy
- Body Health Monitoring (e.g., vitals)
- Cognitive Health (e.g., memory) Monitoring
- Financial Literacy/Management

As we explore each category, I will ask a series of questions. Feel free to interrupt or interject for clarification and additional questions. I am happy to rephrase the question. We want this to be more of a dialog so I encourage you to speak as freely as you please. We want to hear all of your opinions surrounding this space.

General Questions

- Could you walk me through a typical day?
- What technology do you use on a daily basis?
- Have you ever used voice-based technology like amazon alexa, siri, or google home?
  - (if yes) How have you used it?

A social robot is similar to amazon alexa or google home, however, it engages you in a different way than these devices. While these devices are more transactional (you ask them for something, they give it to you), social robots are more relational meaning they use social cues and movement to engage with you in a more social way. For example, when someone is talking to you, you expect them to make eye contact with you. This is one behavior that a social robot may use to interact more relationally with you.

- What are your initial thoughts about social robots?
  - How do you feel about social robots?
  - What role, if any, do you see a social robot playing in your life?

For the next portion of the interview, we will be progressing through six different areas that a social robot could potentially work with you. You may not currently engage in these activities but we ask that you keep an open mind when thinking about the situations. When you are thinking about a robot in these situations, you can imagine the robot as being your ideal robot that is not limited by what technology can currently do in today's world.

We also want to think about this all being in your home.

Medication Adherence

When you hear the words medication adherence, what do you think of?

Is it currently a part of your life?

- (if yes) How do you currently manage your medication adherence?
- (if no) Emphasize imagining the situation.

What are your initial thoughts around social robots assisting with medical adherence?

- How do you see it fitting into your life?
- What roles would you feel comfortable giving to a social robot around medical adherence?
  - Would you want the robot to remind you… [these questions can be followed up with why]
    - when it was time to take your medicine?
    - of the dosage of your medicine?
    - when it was time to get a refill on the medication?
  - Would you like to be able to receive medication instructions and side effects?
    - Would you like to be able to ask questions to the social robot about your medication?
  - Would you like your robot to track your medication and symptoms and provide you information on when it's important to call your doctor?
  - Would you like your robot to have information on how to get in touch with your doctor?
- We've talked about how you could use a robot to help remind you when to take your medicine, tracking your medical information, and connecting with your care team. What are three things that you think would be helpful for the robot to predict or suggest to you based on it knowing and recording your medical information?
- What roles wouldn't you feel comfortable giving to a social robot around medical adherence?
  - What concerns, if any, do you have about a social robot assisting with medical adherence?
  - What benefits, if any, do you imagine with a social robot assisting with medical adherence?

[some participants may be mixed on if they want the robot for medical adherence. Other cases may be on the extremes of wanting the social robot or NOT wanting the social robot. The following questions are for those on either side of the extreme]

- (if very enthusiastic with no concerns) what would make you uncomfortable to have a social robot assist with medical adherence?
- (if very against social robot) what would make you more comfortable to have a social robot assist with medical adherence?

Emotional Wellness Coaching/Mood Sensing and Intervention

When you hear the words emotional wellness coaching in the home, what do you think of?

Is it currently a part of your life?

- (if yes) How do you currently manage your emotional wellness?

- (if no) Emphasize imagining the situation.

What are your initial thoughts about social robots assisting with emotional wellness coaching to help you cope with daily life?

- How do you see it fitting into your life?

- Would you want the robot to… [these questions can be followed up with why]
  - guide you in meditation? yoga?
  - help you appreciate moments in your life more?
  - encourage you to think of blessings in your life or things you are thankful for?
  - encourage you to write gratitude letters to thank people in your life?

Your energy and engagement, how positive or negative you are feeling, and how confident you feel in being able to complete tasks are often connected to wellbeing. If tracking these things may help your wellbeing, would you like a social robot to track…

- How energetic you are? [arousal]
- How positive or negative your mood is? [valence]
- How much in control do you feel you have in life? Do you feel you're juggling different aspects of your life (work, health, social life, family, etc.) well or do you feel overwhelmed by them? [dominance/control; fight or flight]

- Would you like your robot to monitor your overall emotional health and mood long-term?
- We've talked about how you could use a robot to help you be more mindful and cognizant of your emotional wellness and also to track things related to your wellbeing. What are three things that you think would be helpful for the robot to predict or suggest to you based on it knowing and recording your emotional wellbeing?
- Would you like your robot to engage in casual conversation with you?
- Would you like your robot to give you little tokens of appreciation or delightfulness such as jokes, fun facts, surprises, or animations?

- Overall, how do you feel about a social robot engaging you in emotional wellness coaching?
  - What concerns, if any, would you have about this interaction?
  - What benefits, if any, do you imagine from this interaction?

[some participants may be mixed on if they want the robot for emotional wellness coaching. Other cases may be on the extremes of wanting the social robot or NOT wanting the social robot. The following questions are for those on either side of the extreme]

- (if very enthusiastic with no concerns) what would make you uncomfortable to have emotional wellness coaching from a social robot?
- (if very against social robot) what would make you more comfortable to have emotional wellness coaching from a social robot?

Connecting with Others

When you hear the words connecting with others, what do you think of?

How do you currently connect with others?

- (if don't mention any technology) Do you use any technology to connect with others?

What are your initial thoughts about social robots mediating and/or promoting your connections with others?

- How do you see it fitting into your life?

- Would you want the robot to allow you to video-chat with your family and friends?
- Would you want your robot to record video messages to send to another person?
- Would you want your robot to record text messages to send to another person?
- Would you like your robot to remind you to call or talk to friends and family?
- Would you like other people to be able to send messages or call you through your social robot?
- We've talked about how you could use a robot to connect with others. What are three things that you think would be helpful for the robot to predict or suggest to you based on it understanding how you connect with others?

- Overall, how do you feel about a robot mediating and/or promoting your connections with others?
  - What concerns, if any, would you have about this interaction?
  - What benefits, if any, do you imagine from this interaction?

[some participants may be mixed on if they want the robot for connecting them with people in their lives. Other cases may be on the extremes of wanting the social robot or NOT wanting the social robot. The following questions are for those on either side of the extreme]

- (if very enthusiastic with no concerns) what would make you uncomfortable in engaging in mediating/promoting social connectedness with a social robot?
- (if very against social robot) what would make you more comfortable in engaging in mediating/promoting social connectedness with a social robot?

Exercise/Physical Therapy Engagement

When you hear the words exercise and/or physical therapy, what do you think of?

How do you currently manage your exercise and/or physical therapy routines at home?

What are your initial thoughts about social robots promoting or encouraging you in exercise or physical therapy? (can choose exercise if person does not engage in physical therapy)

- How do you see it fitting into your life?

- Would you like your robot to… [these questions can be followed up with why]
  - Guide you through various exercises (perhaps yoga, running, etc.)?
  - Guide you through physical therapy exercises?
  - Track how often you complete your exercises?
  - Track your progress and challenge you to increase your abilities?
  - Share your progress with your friends to keep each other accountable?

We've talked about how you could use a robot to guide you through exercises, track your exercising, and also track your progress. What are three things that you think would be helpful for the robot to predict or suggest to you based on it knowing and recording your exercise and/or physical therapy activities?

- Overall, how do you feel about a social robot promoting or encouraging you with exercise/physical therapy?
  - What concerns would you have about this interaction?
  - What benefits do you imagine from this interaction?

[some participants may be mixed on if they want the robot for exercise/physical therapy. Other cases may be on the extremes of wanting the social robot or NOT wanting the social robot. The following questions are for those on either side of the extreme]

- (if very enthusiastic with no concerns) what would make you uncomfortable in having a social robot engage you in exercise of physical therapy?
- (if very against social robot) what would make you more comfortable in having a social robot engage you in exercise of physical therapy?

Body Signal Monitoring

When you hear the words body signal monitoring, what do you think of? (If struggling can provide examples of heart rate, blood pressure, sleeping pattern, etc.) (If prefer term "vitals" can use that for understandability)

Is it currently a part of your life?

- (if yes) How do you currently measure, keep track, or manage your body signals?
- (if no) Emphasize imagining the situation.

What are your initial thoughts about social robots helping you keep track of your body signals (i.e. heart rate, blood pressure, sleep pattern, etc.)?

- How do you see it fitting into your life?
- What body signals (or vitals) would feel comfortable with the robot tracking?
- If you had a separate device that you would use to record your body signals, would you want your robot to... (*human driven*)
  - Remind you to check your vitals?
  - Conduct a check-in to see if you took your vitals?
  - Track your body signals *that you tell it* long-term?
    - Provide visualizations of your progress over time?
  - Help you be accountable to managing your vitals?
  - Alert you when you should check in with your doctor?
- Would you want your robot to... (*robot driven*)
  - Measure your body signals using sensors in the robot?
  - Track your body signals *that it records* long-term?
  - Alert friends and family when you should check in with your doctor?

- What body signals would you not feel comfortable with the robot tracking?
- Would you want a robot to monitor your safety?
  - Would you want a robot to notify people if you've fallen? Or had a stroke?
- Would you be comfortable wearing a device that would monitor your body signals and communicate to the social robot?
- We've talked about how you could use a robot to help remind you about checking your body signals, holding you accountable, and also roles the robot could take to help you. What are three things that you think would be helpful for the robot to predict or suggest to you based on it knowing and recording your body signals?
  - Would you like the robot to make lifestyle suggestions on your body signal data?
- Overall, how do you feel about a social robot helping you keep track of your body signals?
  - What concerns would you have about this interaction?
  - What benefits do you imagine from this interaction?

[some participants may be mixed on if they want the robot for vital tracking. Other cases may be on the extremes of wanting the social robot or NOT wanting the social robot. The following questions are for those on either side of the extreme]

- (if very enthusiastic with no concerns) what would make you uncomfortable in having a social robot monitor your vitals?
- (if very against social robot) what would make you more comfortable in having a social robot monitor your vitals?

Cognitive Health Monitoring

When you hear the words cognitive health, what do you think of?

Is it currently a part of your life at the moment?

- (if yes) How do you currently manage your cognitive health?
- (if no) Emphasize imagining the situation.

What are your initial thoughts about social robots helping you manage and/or monitor your memory or cognitive health?

- How do you see a social robot assisting with monitoring your memory fitting into your life?
- What memory exercises would you feel comfortable engaging in with the robot?
- Would you want to… [these questions can be followed up with why]
  - Play games with your robot so that you can exercise your memory?
  - Record notes or lists?
  - Remind you of where objects are located?
  - Repeat things it has said?
  - Record your daily activities?
    - Would you want to share these with others?
  - Record stories on the robot to store memories and remember your past?

    - Would you want to share these with others?
- What memory exercises or recordings would you not feel comfortable engaging in with the robot?
- We've talked about how you could use a robot to help engage you in tasks to help with your memory. What are three things that you think would be helpful for the robot to predict or suggest to you based on it knowing and recording your memory progress?
- Overall, how do you feel about a social robot monitor and/or manage your memory?
  - What concerns would you have about this interaction?
  - What benefits do you imagine from this interaction?

[some participants may be mixed on if they want the robot for cognitive health monitoring. Other cases may be on the extremes of wanting the social robot or NOT wanting the social robot. The following questions are for those on either side of the extreme]

- (if very enthusiastic with no concerns) what would make you uncomfortable in having a social robot engage you in memory exercises?
- (if very against robot) what would make you more comfortable in having a social robot engage you in memory exercises?

Financial Literacy

When you hear the words financial literacy, what do you think of?

Is it currently a part of your life at the moment?

- (if yes) How do you currently manage your finances?
- (if no) Emphasize imagining the situation.

What are your initial thoughts about social robots helping you manage your finances?

- How do you see a social robot assisting with finances fitting into your life?

- Overall, how do you feel about a social robot assisting with finances?
  - What concerns would you have about this interaction?
  - What benefits do you imagine from this interaction?

[some participants may be mixed on if they want the robot for financial literacy. Other cases may be on the extremes of wanting the social robot or NOT wanting the social robot. The following questions are for those on either side of the extreme]

- (if very enthusiastic with no concerns) what would make you uncomfortable in having a social robot help you manage your finances?
- (if very against robot) what would make you more comfortable in having a social robot help you manage your finances?

User Feedback

- Thinking of all of these scenarios, would you want the robot to listen to you and adjust it's behavior to best fit you and your needs?
- How would you like to give feedback to the robot so it can fit you better?

    - Surveys? Vocal feedback?

Closing Thank you for participating in today's interview. These are all the questions I had planned for today.

Do you have any additional thoughts in your mind?

- What interaction scenario did you find most intriguing? Why?
- What interaction scenario did you find most appalling or shocking? Why?
- Do you foresee yourself ever having a social robot in your home?
    - What would it be for?
    - What are the barriers?
- What do you believe would be the benefits of having a social robot in your home?
- What do you think are the disadvantages of having a social robot in your home?
- What are some things that you think we should consider when designing social robots?

Do you have any other questions?

Thank you, again, for your time today. Please let us know if you need anything for us or have more ideas. I will be in contact for the next parts of the study.

1.1 Reflection Interview Protocol

Reflection on the Design Guideline Session The last time we met, you participated in the design guideline generation session with our lab and the other participants.

- What did you think about the session?
- What did you find most intriguing? Why?
- What most appalling or shocking? Why?
- What do you believe would be the benefits of the design guideline session?
    - What did you like about the design guideline session?
- What do you think are the disadvantages of the design guideline session?
    - What would you change about the design guideline session?

Getting into Card Sorting Thank you for coming today to participate in our interview. Today, we are recapping the areas we have been exploring and asking some general questions as well.

Before we get into the categories we have been exploring,

- Broadly, after going through the study, what role do you see a social robot playing in your life?

Now, I'll remind you of the seven categories we started investigating:

- Medication Adherence
- Emotional Wellness Coaching/Mood Sensing and Intervention
- Connecting with Others
- Exercise / Physical Therapy
- Body Health Monitoring (e.g., vitals)

- Memory (Cognitive Health (e.g., memory) Monitoring)
- Financial Literacy/Management

As we explore each category, we will open with a set of questions and then do some card sorting.

Medication Adherence

- What are your thoughts around social robots assisting with medical adherence?
  - How do you see it fitting into your life?

*** CARD SORTING HERE ***

- What was going through your mind when you were sorting the cards the way you did?
- Overall, how do you feel about a social robot engaging you in medical adherence?
  - What concerns, if any, do you have about a social robot assisting with medical adherence?
  - What benefits, if any, do you imagine with a social robot assisting with medical adherence?

Emotional Wellness Coaching/Mood Sensing and Intervention

- What are your thoughts about social robots assisting with emotional wellness coaching to help you cope with daily life?
  - How do you see it fitting into your life?

*** CARD SORTING HERE ***

- What was going through your mind when you were sorting the cards the way you did?
- Overall, how do you feel about a social robot engaging you in emotional wellness coaching?
  - What concerns, if any, would you have about this interaction?
  - What benefits, if any, do you imagine from this interaction?

Connecting with Others

- What are your thoughts about social robots mediating and/or promoting your connections with others?
  - How do you see it fitting into your life?

*** CARD SORTING HERE ***

- What was going through your mind when you were sorting the cards the way you did?
- Overall, how do you feel about a robot mediating and/or promoting your connections with others?
  - What concerns, if any, would you have about this interaction?
  - What benefits, if any, do you imagine from this interaction?

Exercise/Physical Therapy Engagement

- What are your thoughts about social robots promoting or encouraging you in exercise or physical therapy? (can choose exercise if person does not engage in physical therapy)
  - How do you see it fitting into your life?

*** CARD SORTING HERE ***

- What was going through your mind when you were sorting the cards the way you did?
- Overall, how do you feel about a social robot promoting or encouraging you with exercise/physical therapy?
  - What concerns would you have about this interaction?
  - What benefits do you imagine from this interaction?

Body Signal Monitoring What are your thoughts about social robots helping you keep track of your body signals (i.e. heart rate, blood pressure, sleep pattern, etc.)?

- How do you see it fitting into your life?
- What body signals (or vitals) would you want the robot tracking?

*** CARD SORTING HERE ***

- What was going through your mind when you were sorting the cards the way you did?
- Overall, how do you feel about a social robot helping you keep track of your body signals?
  - What concerns would you have about this interaction?
  - What benefits do you imagine from this interaction?

Cognitive Health Monitoring

- What are your initial thoughts about social robots helping you manage and/or monitor your memory or cognitive health?
  - How do you see a social robot assisting with monitoring your memory fitting into your life?

*** CARD SORTING HERE ***

- What was going through your mind when you were sorting the cards the way you did?
- Overall, how do you feel about a social robot monitor and/or manage your memory?
  - What concerns would you have about this interaction?
  - What benefits do you imagine from this interaction?

Closing Thank you for participating in today's interview. These are all the questions I had planned for today.

Do you have any additional thoughts in your mind?

- What interaction scenario did you find most intriguing? Why?
- What interaction scenario did you find most appalling or shocking? Why?
- Do you foresee yourself ever having a social robot in your home?
  - What would it be for?
  - What are the barriers?
- What do you believe would be the benefits of having a social robot in your home?
- What do you think are the disadvantages of having a social robot in your home?
- What are some things that you think we should consider when designing social robots?

Interview Reflection

- What did you think of the interview?
- What did you like about the interview?
- What would you change about the interview?
- What did you think about this interview compared to our first interview?

Overall CoDesign Reflection

- What did you think of our codesign process?
- What did you like about our codesign process?
- What would you change about our codesign process?
- How would you like to continue our collaboration?

Do you have any other questions?

Thank you, again, for your time today. Please let us know if you need anything for us or have more ideas. We will continue to be in touch.

2 INITIAL INTERVIEW FINAL DECISION TREES

p01, p02, p03+p04, p05, p06, p07, p08, p09, p11+p12, p13, p14+p15, p18+p19, p20, p21, p22, p23, p25, p27, p29, p30+p28, p31

medication adherence

basic functions

- reminders/medical journal
  - dosage, refill, medication, check-ins, appointments
- doordinator
  - connect to clinics and doctors
- hold users accountable

solvable obstacles

- pre-existing methods
  - refills, side effects
- dustomization
  - reminder frequency and tone
    - no nagging
  - medical information and history
  - tracking and sharing data

barriers

- autonomy
  - does not want to become overly dependent and lose agency
  - retain control
- privacy and security
  - information must be safe
  - have to have trust
- lack of expertise
  - some prefer going to doctor for certain information

main points

Participants find this function to be the most useful, especially for administrative functions such as setting reminders and contacting others. They hope to be able to customize the frequency of alerts, and the amount of medical information/history that the robot offers. The main barriers for the participants are their autonomy, the fear of becoming over-reliant on the robot, and the privacy of user data.

p01, p02, p03+p04, p05, p06, p07, p08, p09, p11+p12, p13, p14+p15, p18+p19, p20, p21, p22, p23, p25, p27, p29, p30+p28, p31

emotional wellness

basic functions

- jokes and fun facts
- emotional support
  - encouragements to socialize
- acts as a pet
  - eompanionship

solvable obstacles

- customization
  - yoga and meditation
  - energy level and mood tracking
  - list of friends to contact
  - gratitude reminders
  - causal conversation
- tone of reminders
  - encouraging
- othering
  - more useful for those alone

barriers

- privacy
  - mood tracking seems too intrusive
- autonomy
  - does not want social interactions to feel like an obligation
  - can choose the pool of people from which the robot can recommend users to contact
- lack of human-ness
  - apprehensive that a robot can have a real relationship

main points

Participants mainly envision the robot supporting emotional wellness by playing a casual role, such as telling jokes to lighten the mood. Some could imagine the robot being able to encourage social connections with others, but many are skeptical that the robot itself could offer real companionship itself due to its lack of humanness. Some also think that this functional category is more useful for those feeling isolated.

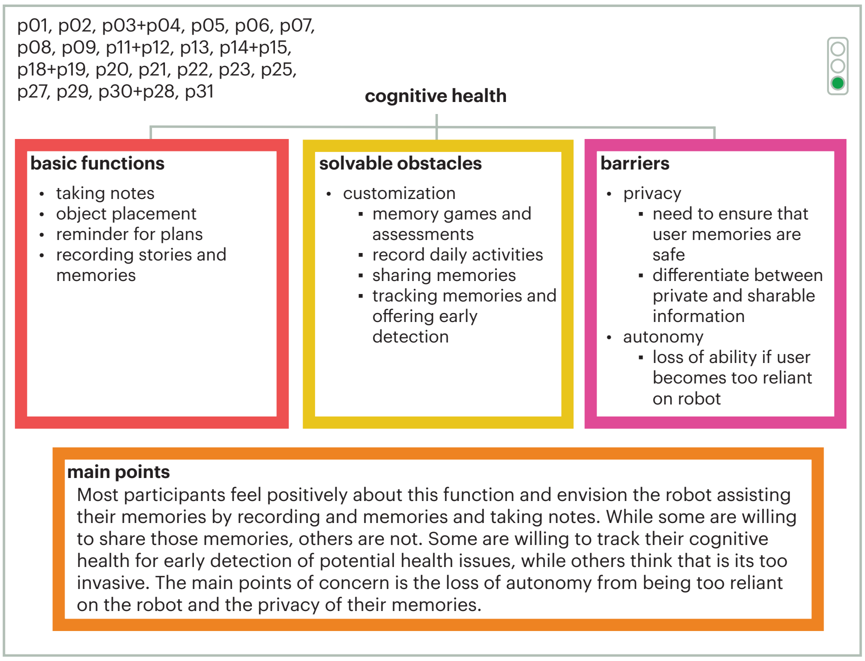
p01, p02, p03+p04, p05, p06, p07, p08, p09, p11+p12, p13, p14+p15, p18+p19, p20, p21, p22, p23, p25, p27, p29, p30+p28, p31
cognitive health
basic functions
taking notes
object placement
reminder for plans
recording stories and memories
solvable obstacles
customization
memory games and assessments
record daily activities
sharing memories
tracking memories and offering early detection
barriers
privacy
need to ensure that user memories are safe
differentiate between private and sharable information
autonomy
loss of ability if user becomes too reliant on robot
main points
Most participants feel positively about this function and envision the robot assisting their memories by recording and memories and taking notes. While some are willing to share those memories, others are not. Some are willing to track their cognitive health for early detection of potential health issues, while others think that is its too invasive. The main points of concern is the loss of autonomy from being too reliant on the robot and the privacy of their memories.

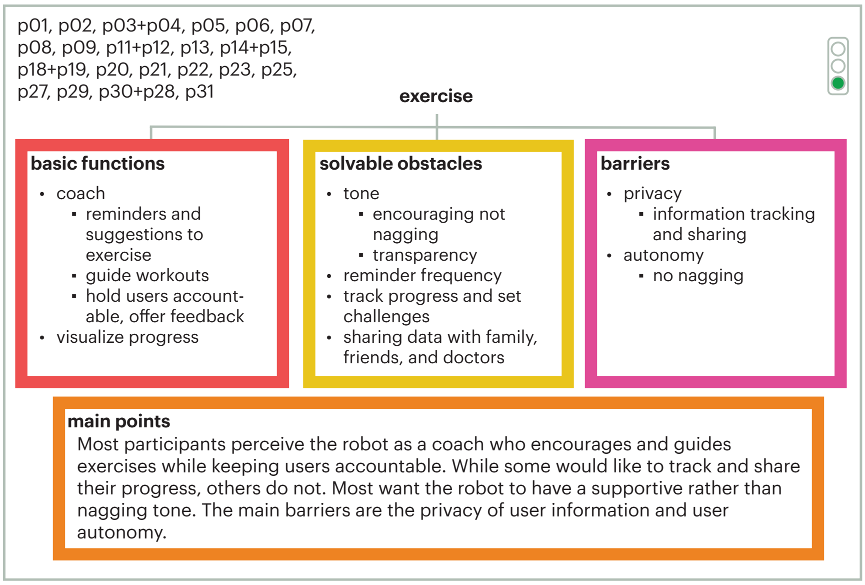
p01, p02, p03+p04, p05, p06, p07, p08, p09, p11+p12, p13, p14+p15, p18+p19, p20, p21, p22, p23, p25, p27, p29, p30+p28, p31
exercise
basic functions
coach
reminders and suggestions to exercise
guide workouts
hold users account-able, offer feedback
visualize progress
solvable obstacles
tone
encouraging not nagging
transparency
reminder frequency
track progress and set challenges
sharing data with family, friends, and doctors
barriers
privacy
information tracking and sharing
autonomy
no nagging
main points
Most participants perceive the robot as a coach who encourages and guides exercises while keeping users accountable. While some would like to track and share their progress, others do not. Most want the robot to have a supportive rather than nagging tone. The main barriers are the privacy of user information and user autonomy.

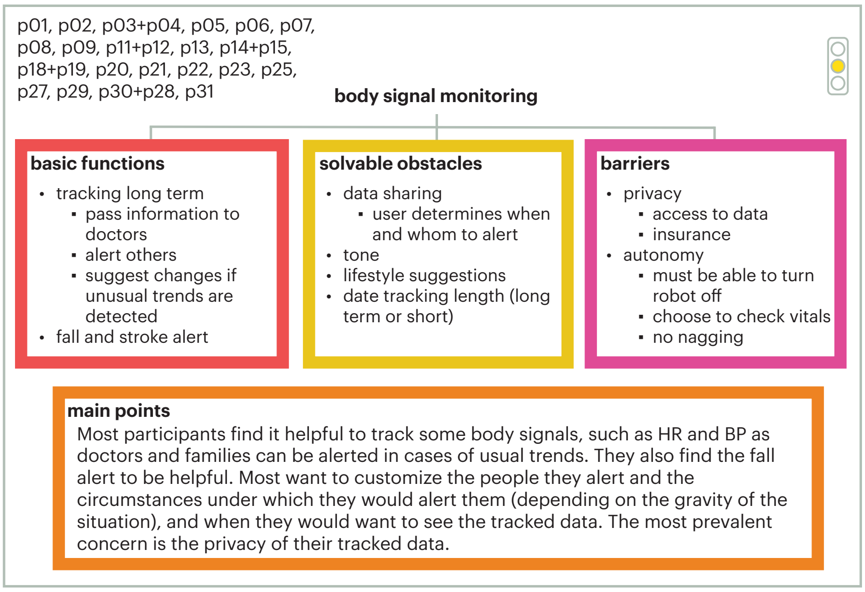

p01, p02, p03+p04, p05, p06, p07, p08, p09, p11+p12, p13, p14+p15, p18+p19, p20, p21, p22, p23, p25, p27, p29, p30+p28, p31
body signal monitoring
basic functions
tracking long term
pass information to doctors
alert others
suggest changes if unusual trends are detected
fall and stroke alert
solvable obstacles
data sharing
user determines when and whom to alert
tone
lifestyle suggestions
date tracking length (long term or short)
barriers
privacy
access to data
insurance
autonomy
must be able to turn robot off
choose to check vitals
no nagging
main points
Most participants find it helpful to track some body signals, such as HR and BP as doctors and families can be alerted in cases of usual trends. They also find the fall alert to be helpful. Most want to customize the people they alert and the circumstances under which they would alert them (depending on the gravity of the situation), and when they would want to see the tracked data. The most prevalent concern is the privacy of their tracked data.

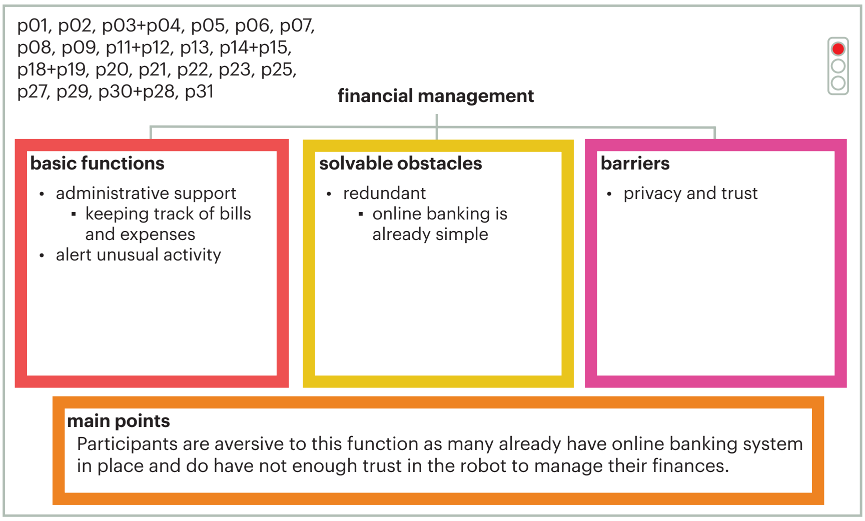

p01, p02, p03+p04, p05, p06, p07, p08, p09, p11+p12, p13, p14+p15, p18+p19, p20, p21, p22, p23, p25, p27, p29, p30+p28, p31
financial management
basic functions
administrative support
keeping track of bills and expenses
alert unusual activity
solvable obstacles
redundant
online banking is already simple
barriers
privacy and trust
main points
Participants are aversive to this function as many already have online banking system in place and do have not enough trust in the robot to manage their finances.

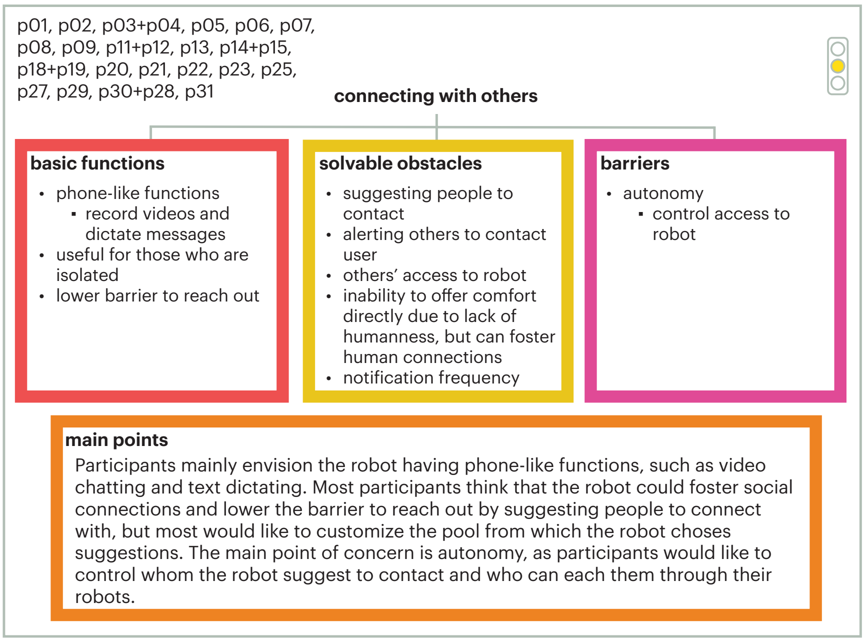
p01, p02, p03+p04, p05, p06, p07, p08, p09, p11+p12, p13, p14+p15, p18+p19, p20, p21, p22, p23, p25, p27, p29, p30+p28, p31
connecting with others
basic functions
phone-like functions
record videos and dictate messages
useful for those who are isolated
lower barrier to reach out
solvable obstacles
suggesting people to contact
alerting others to contact user
others' access to robot
inability to offer comfort directly due to lack of humanness, but can foster human connections
notification frequency
barriers
autonomy
control access to robot
main points
Participants mainly envision the robot having phone-like functions, such as video chatting and text dictating. Most participants think that the robot could foster social connections and lower the barrier to reach out by suggesting people to connect with, but most would like to customize the pool from which the robot choses suggestions. The main point of concern is autonomy, as participants would like to control whom the robot suggest to contact and who can each them through their robots.

3 REFLECTION INTERVIEW FINAL DECISION TREES

p01, p02, p05, p07, p08, p11+p12, p13, p14+p15, p18+p19, p20, p21, p23, p25, p27, p30+p28

medication adherence

basic functions

- reminders
  - take medication, dosage, refills
- provide information
  - side effects, medication information, contacting doctor
- track medication and symptoms

solvable obstacles

- pre-existing methods
  - reminders to take medication, dosage, refills
- alert user to call doctor

barriers

- lack of expertise
  - robot should acquire information from reliable sources
  - medical instructions should come from doctors
- autonomy
  - no nagging
- privacy
  - user data

main points

Overall, participants feel positively about this category. While some participants think that functions that set reminders and provide information are useful, others already have pre-existing methods for those functions. However, many feel that the functions could become more useful as they age. The common concerns are regarding whether the robot is qualified to provide medical instructions and the autonomy and privacy of users.

p01, p02, p05, p07, p08, p11+p12, p13, p14+p15, p18+p19, p20, p21, p23, p25, p27, p30+p28

emotional wellness

basic functions

- monitor emotional health long-term
- little delights

solvable obstacles

- tracking
  - how energetic, positive/negative, in control, overwhelmed the user feels
- encourage appreciation, thankfulness
- gratitude letters
- guide yoga/meditation
- casual conversation

barriers

- privacy
  - could feel intrusive
- autonomy
  - no nagging
- lack of humanness
  - can offer light support but cannot replace human connections

main points

Participants are intrigued by this category. Some participants think that tracking moods and emotions could provide help users become more aware of their mental states, while others think that it is intrusive. Most envision the robot providing light entertainment, such as the little delights, but are skeptical of the robot's ability to form genuine connections. Some participants think that this category would be more useful for those living alone.

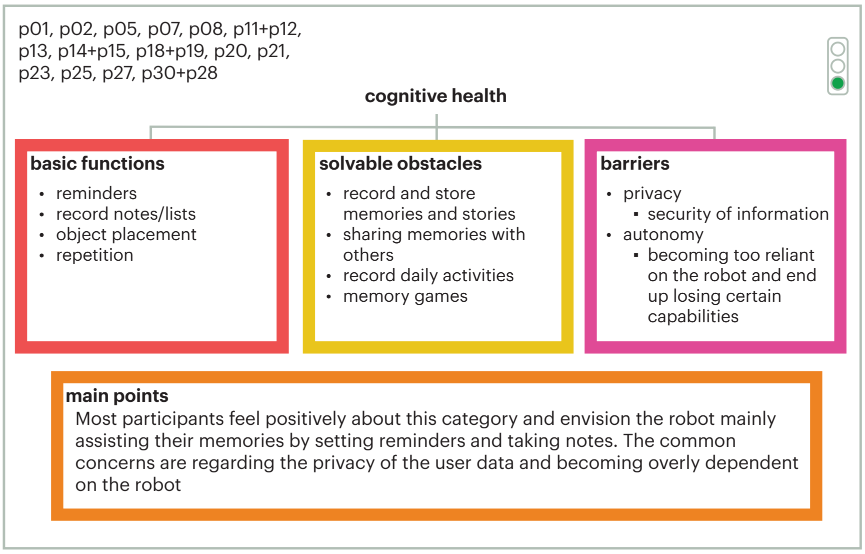
p01, p02, p05, p07, p08, p11+p12,
p13, p14+p15, p18+p19, p20, p21,
p23, p25, p27, p30+p28
cognitive health
basic functions
• reminders
• record notes/lists
• object placement
• repetition
solvable obstacles
• record and store memories and stories
• sharing memories with others
• record daily activities
• memory games
barriers
• privacy
▪ security of information
• autonomy
▪ becoming too reliant on the robot and end up losing certain capabilities
main points
Most participants feel positively about this category and envision the robot mainly assisting their memories by setting reminders and taking notes. The common concerns are regarding the privacy of the user data and becoming overly dependent on the robot

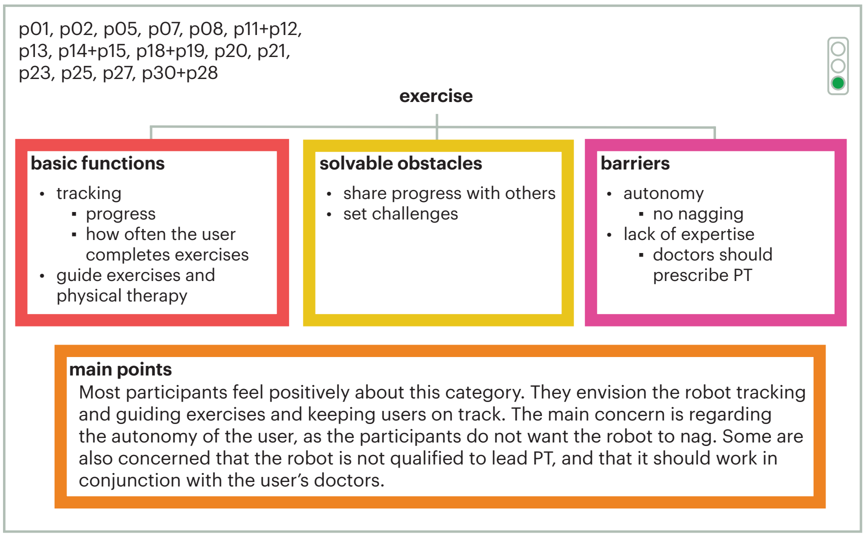
p01, p02, p05, p07, p08, p11+p12,
p13, p14+p15, p18+p19, p20, p21,
p23, p25, p27, p30+p28
exercise
basic functions
• tracking
▪ progress
▪ how often the user completes exercises
• guide exercises and physical therapy
solvable obstacles
• share progress with others
• set challenges
barriers
• autonomy
▪ no nagging
• lack of expertise
▪ doctors should prescribe PT
main points
Most participants feel positively about this category. They envision the robot tracking and guiding exercises and keeping users on track. The main concern is regarding the autonomy of the user, as the participants do not want the robot to nag. Some are also concerned that the robot is not qualified to lead PT, and that it should work in conjunction with the user's doctors.

p01, p02, p05, p07, p08, p11+p12, p13, p14+p15, p18+p19, p20, p21, p23, p25, p27, p30+p28

body signal monitoring

basic functions
- track body signals long-term
- alert user to check-in with doctor
- alert/check-in with user to take vitals
- visualize data
- monitor safety
- stroke/fall alert

solvable obstacles
- personalization
  - connecting to other devices
  - lifestyle suggestions

barriers
- privacy
  - user data
- lack of expertise
  - the robot lacks the medical training of doctors and should not be making diagnoses/sugges-tions
- autonomy
  - user decides who has access to information

main points
Participants are intrigued about this category. They envision long-term body signal tracking to be helpful as it allows the user to notice trends and changes in their health. However, some are concerned about the privacy of their information and the robot's ability to make proper suggestions.

p01, p02, p05, p07, p08, p11+p12, p13, p14+p15, p18+p19, p20, p21, p23, p25, p27, p30+p28

connecting with others

basic functions
- reminding user to contact others

solvable obstacles
- pre-existing methods
  - phone-like functions

barriers
- privacy
- autonomy
  - nagging

main points
Participants could envision the robot encouraging interactions with others by suggesting people to contact. Most participants find phone-like functions to be redundant, but do not mind having them on the robot if they are easier to use. Some participants feel that recommending people to contact would be too nagging.
